# Metabolic engineering of *Lacticaseibacillus paracasei* alleviates post-acidification and reshapes metabolite profiles in probiotic yogurt

**DOI:** 10.1016/j.fochx.2026.104386

**Published:** 2026-09-03

**Authors:** Bohai Li, Longxiang Ye, Rong Dai, Xin Wang, Yongfu Chen

**Affiliations:** a Key Laboratory of Dairy Biotechnology and Engineering, Ministry of Education, Inner Mongolia Agricultural University, Hohhot, Inner Mongolia 010018, China; b Inner Mongolia Key Laboratory of Dairy Biotechnology and Engineering, Inner Mongolia Agricultural University, Hohhot, Inner Mongolia 010018, China; c Key Laboratory of Dairy Products Processing, Ministry of Agriculture and Rural Affairs, Inner Mongolia Agricultural University, Hohhot, Inner Mongolia 010018, China; d China-Mongolia Biomacromolecule Application “Belt and Road” Joint Laboratory, China

**Keywords:** Probiotic yogurt, Post-acidification, Metabolic engineering, *Ldh* knockout, Flavor compounds, Carbon flux

## Abstract

Post-acidification during storage limits the use of multiple probiotics in fermented dairy products. In this study, the *ldh* gene was deleted in *Lacticaseibacillus paracasei* Zhang (LCZ), resulting in 45% reduction in lactic acid production. Co-fermentation of the engineered strain with commercial starters effectively controlled post-acidification, maintained a stable pH (4.5–4.3) over 28 d, and increased production of volatile compounds (acetoin, decanoic acid, and dodecanoic acid) and bioactive metabolites (acetic and succinic acids). Metabolic analysis revealed that *ldh* deletion activated acetoin biosynthesis (750 ng/mL) and redirected carbon flux toward malic, and acetic acids. The modification also promoted amino acid catabolism (L-alanine, L-cysteine, and L-asparagine) to pyruvic acid, enabling nitrogen–carbon co-utilization. This study presents a promising solution for developing stable probiotic dairy products and provides new insights into probiotic metabolic engineering.

## Highlights

• **ldh** deletion reduced probiotic-associated post-acidification in yogurt.
• The **ldh**-deletion strain remained viable without prolonging yogurt fermentation.
• **ldh** deletion reshaped the metabolite profile of probiotic yogurt.
• **ldh** deletion increased flavor-related and bioactive metabolites.

## 1 Introduction

Traditionally, yogurt is produced by fermenting milk with *Streptococcus thermophilus* and *Lactobacillus bulgaricus* in a sterile environment at 36–42 °C for 3–8 h. These bacteria ferment lactose into lactic acid and other compounds, contributing to milk thickening while enhancing its flavor, appearance, texture, and digestibility. Yogurt provides several health benefits, including improved digestion, immune support, and bone health (Pei, Martin, DiMarco, & Bolling, 2017). Additionally, its appealing taste makes it a consumer-friendly vehicle for delivering probiotics, encouraging regular consumption compared with alternatives, such as capsules or powders. The thick matrix of yogurt also protects probiotics from gastric acidity during transit, enhancing their chances of survival until they reach the intestine (Pereira et al., 2024). Several probiotic strains, particularly those from the *genera Lactobacillus and Bifidobacterium*, are commonly incorporated into yogurt. Co-fermentation of starter cultures with probiotics in milk enhances flavor, texture, and functional properties, including inhibition of tumor development (Yang et al., 2020), improvement of digestive or constipation symptoms (Moslemi, Mazaheri Nezhad Fard, Hosseini, Homayouni-Rad, and Mortazavian, 2016), and alleviation of symptoms in patients with irritable bowel syndrome (Jonkers, Penders, Masclee, & Pierik, 2012). Therefore, the development of yogurt products that combine both probiotic and starter cultures has gained growing interest for their enhanced benefits.

Post-acidification, characterized by active growth and continued production of acid, primarily lactic acid, by bacteria, even under refrigerated conditions (4 °C), is a major challenge in fermented dairy products. Excess lactic acid can negatively affect taste and inhibit the activity of added probiotics. Although industrial starter cultures are often selected to minimize lactic acid production at 4 °C, probiotics are typically not selected based on this criterion. Therefore, co-fermentation of probiotics with industrial starter cultures may exacerbate lactic acid production, leading to a reduced yogurt shelf life. This limitation hinders the application of several beneficial probiotic strains for commercial yogurt production. For example, in our previous studies, we demonstrated the potential of *Lacticaseibacillus paracasei* Zhang (LCZ), a probiotic strain, to lower blood lipid levels (Zhong, Zhang, Du, Meng, & Zhang, 2012), alleviate inflammation (Zhu et al., 2021), enhance acid production, and shorten fermentation time. In contrast, LCZ also accelerates post-acidification of yogurt and shortens its shelf life when fermented with starter cultures (Bai et al., 2020). Therefore, developing an effective strategy to mitigate the probiotic-induced post-acidification of yogurt remains a crucial challenge.

Gene editing technology has emerged as a powerful tool for precisely modifying probiotic genomes to optimize their properties. Unlike conventional fermentation control methods that rely on environmental adjustments and may show batch-to-batch variations, metabolic engineering through gene editing offers distinct advantages of precise, stable, and heritable modifications at the genetic level. This approach has shown promise in enhancing probiotic traits in several species, including *Lactobacillus* and *Bifidobacterium* (Zuo & Marcotte, 2021). The lactate dehydrogenase (LDH) encoded by the *ldh* gene is a key enzyme that catalyzes the reduction of pyruvate to lactic acid, making it a prime target for metabolic engineering (Dai et al., 2020). Targeted knockout of *ldh* in probiotic strains may reduce or block lactic acid production, thereby offering a potential solution for controlling post-acidification in probiotic yogurt products.

In this study, we employed prophage-derived recombinase-mediated recombineering to knockout *ldh* in the LCZ genome with the aim of developing a modified LCZ strain with reduced lactic acid production capabilities, thus minimizing post-acidification yogurt. Additionally, the flavor and metabolic profiles of yogurt fermented with both the *ldh* knockout (Δ*ldh*) and the original strains, along with starter cultures during fermentation and storage, were compared and analyzed. Our findings provide a promising approach to preserve yogurt quality during storage while maintaining probiotic health benefits.

## 2 Methods

### 2.1 Strains and culture conditions

The wild-type LCZ strain, an unmodified parental strain originally isolated from traditional home-made koumiss, was obtained from the Key Laboratory of Dairy Biotechnology and Engineering, Inner Mongolia Agricultural University. It was used for construction of the Δ*ldh* mutant. Its complete genome sequence was retrieved from the NCBI database (NCBI txid: 498216). The starter culture YoFlex® Mild 1.0, containing *Lactobacillus delbrueckii* subsp. *bulgaricus* and *Streptococcus thermophilus*, was purchased from Chr. Hansen (Copenhagen, Denmark). According to the supplier's product information, this starter culture exhibits very low post-acidification. Wild-type LCZ and its mutants were cultured in De Man Rogosa Sharpe (MRS) broth (Oxoid) at 37 °C for 24 h. Bacterial cells were harvested by centrifugation at 8000 ×*g* for 15 min at 4 °C and subsequently resuspended in sterile saline. Bacterial growth was monitored by measuring the optical density of the cultures at 600 nm (OD_600_) using the Bioscreen C system (Growth Curves AB Ltd., Helsinki, Finland).

### 2.2 Plasmids and DNA manipulation

Plasmids pMSP456 and pMSPCre were obtained from the State Key Laboratory of Microbial Technology, Shandong University. pMSP456 was used for co-expression of the prophage-derived recombinase *LCABL 13040–50-60*, while plasmid pMSPCre was used to express *Cre* recombinase (Xin, Guo, Mu, & Kong, 2017). pNZ5319, which contains *lox66* and *lox71* sites, was purchased from NovoPro Biosciences (Shanghai, China). *Escherichia coli* DH5α was used as cloning host. All *E. coli* strains carrying plasmids were cultivated in Luria-Bertani broth (LB) at 37 °C with aeration. When necessary, antibiotics were added at the following concentrations: 5 μg/mL erythromycin (Sigma-Aldrich, MO, USA) or chloramphenicol (Sigma-Aldrich) for *Lactobacillus* strains, and 250 μg/mL erythromycin and 35 μg/mL chloramphenicol for *E. coli* strains.

Genomic DNA from LCZ was extracted using the TIANGEN DNA extraction kit (Beijing, China), following the manufacturer's instructions. Plasmids pMSP456γ, pMSPCre, and pNZ5319 were isolated from *E. coli* using an Axygen Plasmid Extraction Kit (Axygen Scientific Inc., CA, USA). *ldh* was identified in the LCZ genome using Prokka and NCBI BLAST; The sequences are provided in Table S1. Primers used in this study are listed in Table S2. To construct the linear donor *ldh* disruption cassette, the *lox66* and *lox71* sites, along with the chloramphenicol resistance gene (*cat*), were amplified from the plasmid pNZ5319 via PCR using the primers catF and catR, resulting in a *lox66*-*cat*-*lox71* dsDNA fragment. To create homology arms, approximately 1000 bp of upstream and downstream sequences flanking *ldh were* amplified from LCZ chromosomal DNA using the following primers: *ldh*-uF/*ldh*-uR for the upstream arms and *ldh*-dF/*ldh*-dR for the downstream arms. The *lox66*-*cat*-*lox71* dsDNA fragment was then assembled with upstream and downstream homology arms using overlap extension PCR, generating the final linear donor *ldh* disruption cassettes. PCR amplifications were performed using PrimeSTAR® HS DNA polymerase (Takara Bio Inc., Shanghai Branch, Shanghai, China).

### 2.3 Prophage-derived recombinases mediated gene deletion in LCZ

Plasmid pMSP456 was introduced into LCZ via electroporation. Briefly, 200 μL of an overnight LCZ culture was inoculated into 5 mL of MRSG (MRS supplemented with 1% glycine and 0.75 M sorbitol) medium to an OD_600_ of 0.6–0.8. The culture was then chilled at 4 °C for 30 min and centrifuged at 5000 ×*g* for 10 min at 4 °C. The harvested cells were washed twice with 5 mL of ice-cold 10% glycerol and resuspended to prepare electrocompetent cells. For electroporation, 40 μL of competent cells was mixed with 1 μg of plasmid DNA and subjected to electroporation at 2.0 kV, 200 Ω resistance, and 25 μF capacitances using an electroporator. The transformed cells were recovered in 1 mL of SMRS medium (MRS supplemented with 0.1 M MgCl_2_ and 0.5 M sucrose) and incubated at 37 °C for 2 h before plating on MRS agar containing erythromycin for selection.

For *ldh* deletion, the recombinant strain LCZ-456 (LCZ carrying pMSP456) was activated in MRS broth with erythromycin. A 200 μL aliquot of the overnight culture was inoculated into 5 mL of MRSG and incubated at 37 °C until the OD_600_ reached 0.25–0.30. Recombinase expression was induced by addition of nisin (Sigma-Aldrich). Upon reaching an OD_600_ of 0.6–0.8, competent cells were prepared, mixed with 1 μg of the linear donor *ldh* disruption cassette, and electroporated as described above. Transformed cells were then plated on MRS agar containing erythromycin and chloramphenicol for selection. To remove the selectable marker (*lox66*-*cat*-*lox71* fragment), mutant colonies were streaked onto an MRS plate without erythromycin to facilitate the curing of pMSP456. Plasmid-free mutants were subsequently electroporated with pMSPCre as described above. At an OD_600_ of 0.4–0.8, Cre recombinase expression was induced by adding 10 ng/mL nisin for 24 h. The cells were then plated on MRS plates containing erythromycin, and marker-free mutants were identified using colony PCR. Finally, pMSPCre was cured following the method used for pMSP456γ, yielding a mutant strain free of both plasmids and selectable markers.

### 2.4 Yogurt manufacture

Whole milk powder (11% *w*/*v*; per 100 g: 39.1 g lactose, 26.8 g fat, and 25.0 g protein) and sucrose (6.5% *w*/*v*) were food-grade materials that had been screened for pathogenic bacteria before purchase and were confirmed to be free of detectable pathogenic bacteria. They were dissolved in distilled water and hydrated at 50 °C for 30 min. The mixture was homogenized using an SPX APV2000 homogenizer (Beijing, China) at 63 °C and 20 MPa, followed by heating at 95 °C for 25 min. The milk was then cooled to 37 °C for inoculation. Fermented yogurt was divided into three groups: commercial starter culture group (SC), commercial starter culture co-fermented with LCZ (SC + LCZ), and commercial starter culture co-fermented with *ldh* knockout LCZ (SC + Δ*ldh*). The initial viable counts of LCZ and Δ*ldh* strain were standardized to 1 × 10^7^ CFU/mL, and starter culture was added following the manufacturer's instructions (0.3 g/L). Fermentation was carried out in 250 mL sterile bottles (sterilized at 121 °C for 30 min), each containing 200 mL of inoculated milk at 37 °C without agitation. Once the pH reached 4.5, the yogurt was transferred to 4 °C for post-fermentation storage for 28 d. Samples were collected at pH 4.5 and at various time points throughout storage for further analysis.

### 2.5 Determination of pH, TA, and viable cell counts

The pH of the yogurt samples was measured using a pH meter (FE20; Switzerland). Titratable acidity (TA) was measured by mixing 5.0 g of the yogurt sample with 40 mL of hot distilled water (boiled for 15 min and cooled before use). The mixture was then titrated with 0.1 mol/L standardized sodium hydroxide solution to pH 8.1, using 0.5% phenolphthalein as the pH indicator. To determine viable counts of the supplemented probiotic strains, yogurt samples were serially diluted and spread-plated on MRS agar supplemented with 0.5 μg/mL vancomycin. Vancomycin inhibited the commercial starter strains, *Lactobacillus delbrueckii* subsp. bulgaricus and *Streptococcus thermophilus*, but did not inhibit LCZ or its mutant strains. Plates were incubated anaerobically at 37 °C for 48 h, and the total colony counts were enumerated to determine the total viable counts.

### 2.6 Determination of volatile organic compounds (VOCs) using GC-MS

VOCs in the yogurt samples were analyzed using gas chromatography–mass spectrometry (GC-MS; Agilent 8890 GC coupled with an Agilent 7000D GC/MS system (Agilent, USA). For each analysis, 20 mL yogurt was transferred into a 100 mL headspace vial (Agilent). VOCs were extracted using solid-phase microextraction (SPME). The samples were incubated at 50 °C for 1 h before exposing a 120 μm DVB/CWR/PDMS fiber (Agilent) to the sample headspace for 15 min at 60 °C. After extraction, the VOCs were desorbed in the GC injector at 250 °C for 5 min in splitless mode. Helium was used as the carrier gas at a flow rate of 1.2 mL/min. The injector temperature was maintained at 250 °C. The oven temperature was programmed to start at 40 °C (held for 3.5 min), increase at 10 °C/min to 100 °C, followed by an increase of 7 °C/min to 180 °C 25 °C/min to 280 °C (held for 5 min). Mass spectra were recorded in electron impact (EI) ionization mode at 70 eV. The quadrupole mass detector, ion source, and transfer line temperatures were set at 150, 230, and 280 °C, respectively. The scan mode was set to SCAN with a solvent delay of 5 min and scanning mass spectra in the range of *m*/*z* 50–500 at 1-s intervals. VOCs were quantified using the widely targeted volatilomics (WTV) method (H. Yuan et al., 2022) and identified by comparing their mass spectra against the local MWGC library from Metware Biotechnology Co., Ltd. (Wuhan, China). Quality control (QC) samples, prepared as a pooled mixture of all samples, were used to monitor reproducibility. A QC sample was analyzed every 10 samples to ensure analytical consistency.

### 2.7 Determination of non-volatile organic compounds (non-VOCs) using LC-MS

For non-VOC profiling, samples were prepared as follows: 10 mL yogurt was centrifuged at 5000 ×*g* at 4 °C for 10 min. The supernatant was extracted with ice-cold acetonitrile (Merck, Germany) by vortexing for 2 min. Insoluble substances, such as proteins, were removed by centrifugation at 12,000 ×*g* for 10 min. The supernatant was concentrated under vacuum using a Genevac evaporator (EZ 2.3, SP Scientific, New York, USA) and resuspended in 500 μL of a 50% (*v*/v) acetonitrile solution. The samples were then centrifuged at 12,000 ×*g* for 15 min, and the supernatant was filtered through a 0.22 μm microporous membrane before LC-MS analysis using a SCIEX Triple TOF 6600+ mass spectrometer coupled with an ExionLC™ AD liquid chromatography system (AB SCIEX).

The LC parameters were set as follows: column temperature, 40 °C; mobile phase A, ultrapure water with 0.1% formic acid; and mobile phase B, methanol with 0.1% formic acid. The gradient elution program was: 0–1.5 min, 5% B; 1.5–15 min, 5–100% B; 15–18 min, 100% B; 18–19 min, 100–5% B; 19–20 min, 5% B. The flow rate was 0.4 mL/min, using an ACQUITY UPLC HSS T3 C18 column (Waters, USA). The MS parameters were set as follows: curtain gas, 30 psi; ion source gases 1 and 2, 55 psi; source temperature, 550 °C; ion spray voltage, 5500 V (positive mode) and − 4500 V (negative mode); declustering potential, 60 V (positive mode) and − 60 V (negative mode). The collision energies were 20, 35, and 50 eV (positive) and − 20, −35, and − 50 eV (negative), respectively. Data acquisition was performed using information-dependent acquisition (IDA) with dynamic background subtraction. Raw data were processed using the Progenesis QI software (Waters Corp.), including peak extraction, alignment, retention time correction, and deconvolution. Feature identification was performed by searching for MS/MS spectrum matches against both local and public databases. Metabolic networks were constructed using the Search Tool for Interactions of Chemicals (STITCH) to illustrate the relationships between differential metabolites and their associated genes (Szklarczyk et al., 2016). The minimum required interaction score was set to 0.4. The obtained results were downloaded and visualized by the Cytoscape software (v3.10.3).

### 2.8 LC-MS/MS-MRM-based targeted metabolomics analysis

The metabolites of interest were verified by targeted metabolomic analysis using reference standards. All the reference standards were purchased from Sigma-Aldrich. Exometabolites and endometabolites were extracted as follows: cell cultures (2 mL) at the late exponential growth phase (24 h) were collected. Extraction was conducted using a pre-chilled (−80 °C) solvent mixture consisting of methanol: dichloromethane: ethyl acetate (1:2:3, *v*/v/v). The solvent mixture was then added to the samples at a sample/solvent ratio of 1:5. After vigorous vortexing for 2 min, the samples were frozen at −80 °C for 30 min, thawed in an ice bath, and sonication at 20–30 kHz for 5 min. The samples were then centrifuged at 10,000 ×*g* for 10 min at 4 °C. The supernatant, containing both exometabolites and endometabolites, was collected and concentrated using a vacuum rotary evaporator at 30 °C. The dried extracts were reconstituted in 2 mL water/methanol/acetonitrile (2:1:1, *v*/v/v), followed by centrifugation at 10,000 ×*g* for 5 min. Finally, the supernatant was filtered through a 0.22 μm microporous membrane filter prior to LC-MS/MS-MRM analysis.

Owing to the high polarity of pyruvic acid, lactic acid, acetoin, acetic acid, citric acid, and oxaloacetic acid, which prevent effective retention on a C18 column, a derivatization step was performed before detection (Meng et al., 2021). Briefly, 100 μL of the filtered supernatant was mixed with 50 μL of 175 mM 3-nitrophenylhydrazine (3-NPH) in 75% aqueous methanol, 50 μL of 105 mM 1-ethyl-3-(3-dimethylaminopropyl) carbodiimide (EDC) in methanol, and 50 μL of 2.5% pyridine in methanol. The reaction mixture was incubated at 37 °C for 30 min and then evaporated to dryness. The derivatized metabolites were reconstituted in methanol and diluted with three volumes of water to a final volume of 50 μL. All derivatization reagents (3-NPH, EDC, and pyridine) were purchased from Sigma-Aldrich.

Chromatographic separation was performed using an ExionLC™ AD system equipped with an HSS T3 column (2.1 mm × 150 mm, 1.8 μm; Waters, Milford, MA, USA). The mobile phases consisted of (A) 0.1% formic acid in ultrapure water and (B) 0.1% formic acid in methanol. The gradient elution program was as follows: 0.0–1.0 min, 5% B; 1.0–6.5 min, 5–100% B; 6.5–9.5 min, 100% B; 9.5–11.0 min, 100–5% B; 11.0–12.0 min, 5% B. The flow rate was maintained at 0.4 mL/min and the column temperature was set at 40 °C. Mass spectrometric analysis was performed using a SCIEX QTRAP 6500+ system operating in multiple reaction monitoring (MRM) mode. The optimized ion acquisition parameters for all the detected metabolites are listed in Table S3.

### 2.9 Statistical analysis

All experiments in this study were repeated three times in parallel to ensure data reliability. Differential and multivariate data analyses were performed using MetaboAnalyst 5.0 (https://www.metaboanalyst.ca/), including data filter, normalization, analysis of variance (ANOVA), principal component analysis (PCA), partial least squares-discriminant analysis (PLS-DA), and pathway analysis. Data visualization plots, including box plots, bar charts, and heatmaps, were generated using ‘ggpubr,’ ‘ggplot2,’ and ‘pheatmap’ packages using R software (v 4.0.3). Wilcoxon tests were performed to evaluate significant differences between the groups. To reduce the false discovery rate (FDR), *P*-values were corrected using the Benjamini–Hochberg correction. The experimental designs and potential biological mechanisms elucidated in our study were created using Adobe Illustrator (v 29.3) software.

## 3 Results

### 3.1 Genetic disruption of ldh reduced acid production and inhibited post-acidification

**Fig. 1:**
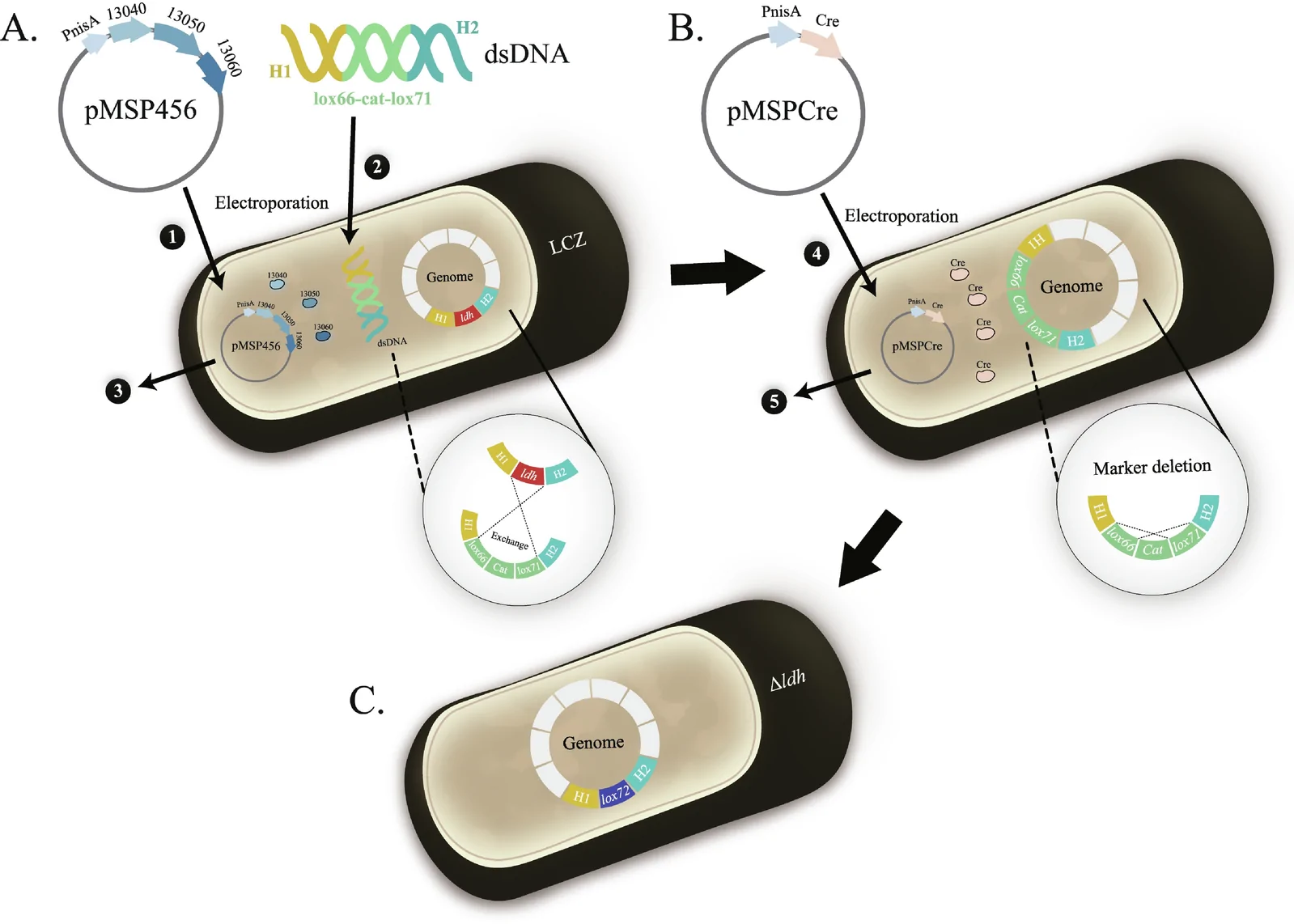
Schematic representation of prophage-derived recombinase-mediated recombineering in *Lacticaseibacillus paracasei* Zhang (LCZ). (A) The pMSP456 plasmid carrying the recombinase operon (LCABL_13,040–50-60) and a lox66-cat-lox71 cassette flanked by homologous arms (H1 and H2) were introduced into LCZ by electroporation. The cassette was integrated by homologous recombination to replace *ldh*, followed by plasmid curing. (B) Cre recombinase-expressing pMSPCre was introduced by electroporation and catalyzed excision of the chloramphenicol-resistance gene (cat) between lox66 and lox71. (C) The resulting Δ*ldh* strain retained a lox72 scar sequence at the edited locus.

**Fig. 2:**
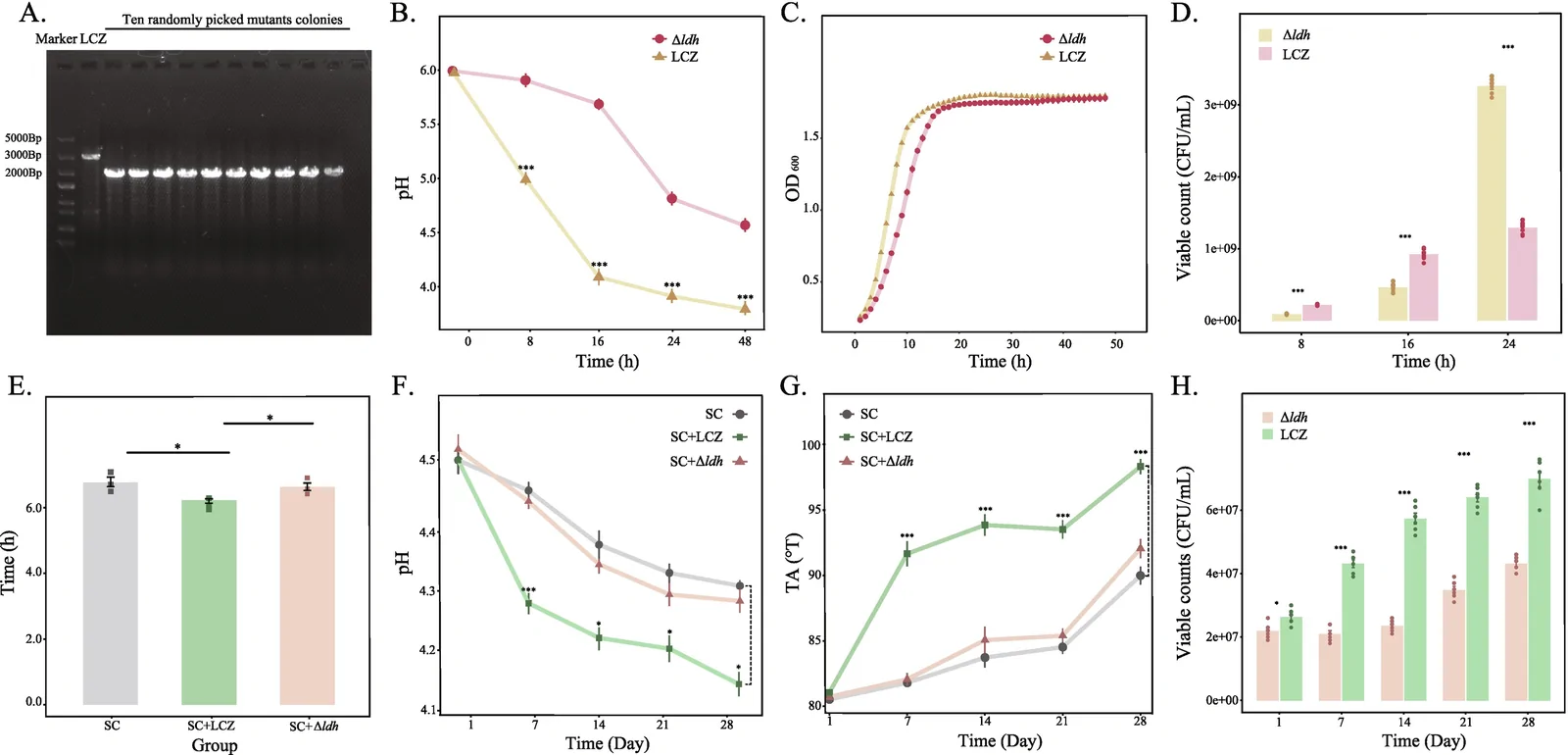
Phenotypic characterization and fermentation properties of the Δ*ldh* strain. (A) PCR verification of *ldh* deletion in ten randomly selected mutant colonies. (B) pH changes in cultures of wild-type LCZ and the Δ*ldh* strain during 48 h in MRS medium. (C) Growth curves of wild-type LCZ and the Δ*ldh* strain, measured as optical density at 600 nm (OD_600_), over 48 h. (D) Viable cell counts of wild-type LCZ and the Δ*ldh* strain during 24 h of culture in MRS medium. (E) Time required for the SC, SC + LCZ, and SC + Δ*ldh* yogurt groups to reach pH 4.5. (F) pH changes and (G) titratable-acidity (TA) changes in the three yogurt groups during 28 d of storage. (H) Viable counts of the supplemented probiotic strains in SC + LCZ and SC + Δ*ldh* during storage. The SC group is not shown in panel H because it did not contain a supplemented probiotic strain. In panels F and G, statistical significance was assessed relative to the SC group; only significant differences are indicated by *, **, and *** (*P* < 0.05, *P* < 0.01, and *P* < 0.001, respectively). In panel E, horizontal connecting lines indicate pairwise comparisons.

The procedure for prophage-derived recombinase-mediated dsDNA recombineering (Xin et al., 2017b) to knockout *ldh* in the LCZ genome is shown in Fig. 1A. pMSP456 carrying a λ Red-like recombinase operon (LCABL_13,040–50-60, analogs of Redγ, Redα, and Redβ) was first electroporated into LCZ (step 1). Subsequently, a dsDNA substrate containing a *lox66-cat-lox71* cassette with homologous arms (H1 and H2) targeting the *ldh* locus was introduced (step 2). The recombination machinery facilitated exchange between the cat cassette and *ldh* in the LCZ genome, resulting in chloramphenicol-resistant recombinants. After curing pMSP456 (step 3), pMSPCre-expressing Cre recombinase under the *PnisA* promoter was transformed (step 4, Fig. 1B) to catalyze site-specific recombination between *lox66* and *lox71* sites. Following pMSPCre curing (step 5), a marker-less Δ*ldh* was obtained (Fig. 1C). *Ldh* excision was confirmed by PCR amplification of ten randomly selected colonies. Primers located 1020 bp upstream and 941 bp downstream of *ldh* (981 bp) generated the expected amplicons of 2001 bp for Δ*ldh* and 2942 bp for the wild-type strains (Fig. 2A), confirming successful generation of the Δ*ldh* strain. The pH of the MRS medium used for the Δ*ldh* strain was significantly higher (*P* < 0.01) than that of the wild-type strain after 48-h of culture (Fig. 2B). The Δ*ldh* strain exhibited a slower growth rate and lower viable count during the logarithmic phase (Fig. 2C and D). Nevertheless, the viable cell count of the Δ*ldh* strain was significantly higher than that of the wild-type strain in the stationary phase at 24 h (*P* < 0.01; Fig. 2D).

Yogurt fermentation experiments showed that co-fermentation with LCZ (SC + LCZ) significantly shortened the time required to reach pH 4.5 compared with the commercial starter culture alone (SC) (*P* < 0.05; Fig. 2E). In contrast, the fermentation time of the SC + Δ*ldh* group did not differ significantly from that of the SC group (*P* > 0.05; Fig. 2E). During storage, LCZ-supplemented yogurt exhibited significant post-acidification (*P* < 0.05), with the pH decreasing to approximately 4.15, and TA increasing to approximately 100°T after 28 d (Fig. 2F and G). In contrast, yogurt containing Δ*ldh* showed no significant changes in pH or TA compared to the commercial starter. The viable counts in both LCZ- and Δ*ldh-*supplemented yogurt increased during storage, with SC + LCZ displaying significantly higher counts than SC + Δ*ldh* (Fig. 2H). No colonies were observed when samples from the SC group were plated on the same vancomycin-containing MRS agar.

### 3.2 Significant flavor shifts occurred in LCZ-co-fermented yogurt, while ldh-knockout LCZ yielded milder changes

**Fig. 3:**
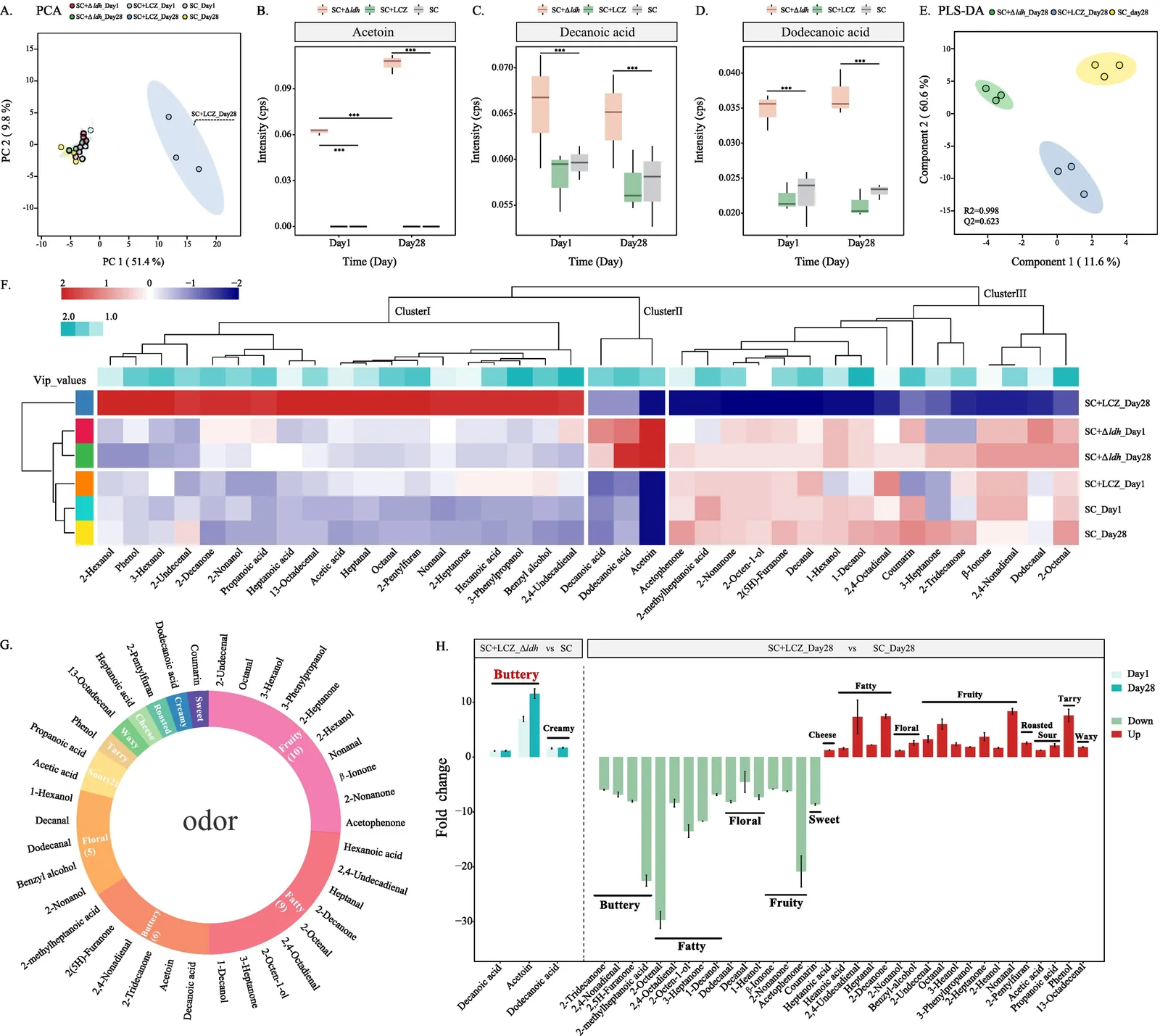
Volatile organic compound (VOC) profiles of yogurt samples from the SC, SC + LCZ, and SC + Δ*ldh* groups. (A) Principal component analysis (PCA) of VOC profiles. (B–D) Relative intensities of acetoin, decanoic acid, and dodecanoic acid, respectively. (E) Partial least squares-discriminant analysis (PLS-DA) of VOC profiles. (F) Heatmap of 38 differential VOCs selected using variable importance in projection (VIP > 1) and FDR < 0.05. (G) Odor-category assignment of the selected VOCs. (H) Fold-change comparisons of VOCs for SC + Δ*ldh* versus SC and SC + LCZ on day 28 versus SC on day 28, grouped by odor category. Group labels are used consistently throughout: SC, SC + LCZ, and SC + Δ*ldh*.

Analysis of the VOC profiles of yogurt fermented with SC + LCZ, SC + Δ*ldh*, and SC alone using GC–MS at the end of fermentation (day 1) and after storage (day 28) identified 567 volatile compounds in all yogurt samples. The metabolomic data from all QC samples exhibited a high level of consistency in the overlay of total ion current (Fig. S1), indicating the robust stability of the GC–MS system. PCA showed that SC + Δ*ldh* (days 1 and 28), SC + LCZ (day 1), and SC alone (days 1 and 28) clustered closely together, indicating minimal changes in their volatile profiles (Fig. 3A). In contrast, the SC + LCZ (day 28) samples were clearly separated from all other samples along PC1 (accounting for 51.4% of the variation), suggesting a substantial shift in the volatile composition of this group after storage. ANOVA revealed that only three compounds—acetoin, decanoic acid, and dodecanoic acid—differed significantly across the yogurt groups on day 1 (FDR < 0.05; Fig. S2A). The intensity of acetoin in the SC + Δ*ldh* group was significantly higher than that in the SC and SC + LCZ groups (FDR < 0.05) on day 1 (Fig. 3B), whereas acetoin continued to increase in the SC + Δ*ldh* group until day 28, with levels remaining near zero in the SC and SC + LCZ groups. The intensities of the decanoic acid and dodecanoic acid (Fig. 3C and D) were also significantly higher (FDR < 0.05) in the SC + Δ*ldh* group than that in the SC or SC + LCZ groups at both time points, with no significant changes observed between days 1 and 28.

ANOVA across yogurt groups on day 28 identified 152 VOCs with significant differences among the samples (Fig. S2B). To refine the selection of the key differential metabolites, a PLS-DA model was constructed (Fig. 3E). The first two components of the model accounted for 72% of the total variance, indicating that metabolomic differences among the groups were well represented. The accuracy and reliability of the PLS-DA model were validated through cross-validation, yielding a sum of squares (R^2^) of 0.998 and a cross-validated R^2^ (Q^2^) of 0.623, with significant results (*P* < 0.05), demonstrating the high validity and robustness of the model. A total of 38 key differential metabolites were identified (Table S4) based on their variable importance in projection (VIP) values (>1, PLS-DA) and significance (FDR < 0.05, Kruskal–Wallis test) (Fig. 3F). Hierarchical clustering of the 38 key metabolites divided yogurt samples into three main branches. SC + LCZ (day 1), SC (day 1), and SC (day 28) were clustered together, indicating similar profiles. SC + Δ*ldh* (day 1) and SC + Δ*ldh* (day 28) were grouped into a second branch, suggesting a consistent volatile composition over storage. SC + LCZ (day 28) clustered independently, in line with the PCA findings, which highlighted its distinct profile after storage. Metabolite clustering revealed that 19 compounds in Cluster I and 16 in Cluster III were enriched and depleted, respectively, in the SC + LCZ group (day 28), explaining its unique composition. Three metabolites in Cluster II—acetoin, decanoic acid, and dodecanoic acid—were enriched in SC + Δ*ldh* (day 1) and SC + Δ*ldh* (day 28), closely matching earlier observations of their distinct profiles (Fig. 2B–D).

An investigation of the odor attributes (Fig. 3G) showed that the 38 key metabolites spanned 10 distinct aroma categories: fruity, floral, buttery, fatty, sour, tarry, waxy, cheese, roasted, creamy, and sweet. Fold-change comparisons (Fig. 3H) further illustrated these changes: in the SC + Δ*ldh* group, acetoin (a buttery-flavored compound) showed the largest increase on both days 1 and 28, whereas in SC + LCZ (day 28), most buttery-associated metabolites were markedly reduced. In addition, other aroma contributors related to fatty, floral, and fruity notes also shifted substantially in SC + LCZ (day 28). Notably, phenol, a tarry flavor compound, was significantly increased in SC + LCZ (day 28). Phenol is considered undesirable due to its negative impact on flavor and its reported cytotoxicity at high concentrations. Its formation may be associated with microbial tyrosine metabolism or raw material- and environment-derived sources.

### 3.3 Ldh-knockout LCZ yogurt exhibited distinct non-volatile metabolite profiles during fermentation and storage

**Fig. 4:**
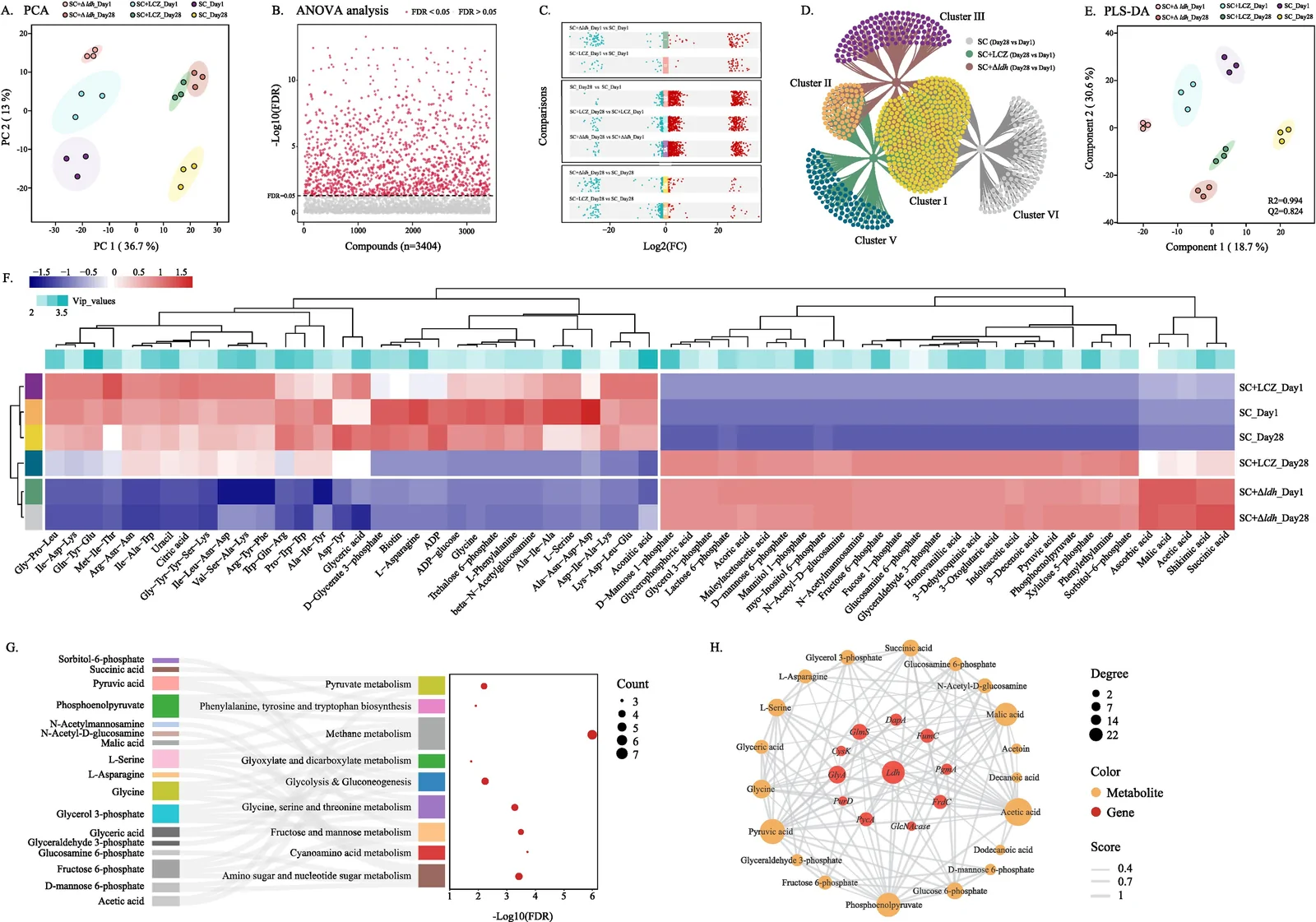
Non-volatile metabolite profiles of yogurt samples from the SC, SC + LCZ, and SC + Δ*ldh* groups. (A) Principal component analysis (PCA) of non-volatile metabolite profiles. (B) ANOVA of metabolites across groups. (C) Differential metabolites between groups (FDR < 0.05 and |log2 fold change| > 1); red and blue dots denote higher and lower relative abundance, respectively. (D) Venn analysis of storage-associated metabolite changes. (E) Partial least squares-discriminant analysis (PLS-DA). (F) Heatmap of key differentially abundant metabolites selected using VIP > 2 and FDR < 0.05. (G) Pathway enrichment analysis; dot size represents the number of mapped metabolites. (H) Knowledge-based metabolite–gene interaction network. Node size indicates network connectivity and edge thickness indicates the confidence score. Red nodes represent genes and yellow nodes represent metabolites. Abbreviations: LDH, lactate dehydrogenase; FumC, fumarate hydratase; PgmA, phosphoglucomutase; FrdC, fumarate reductase; GlcNAcase, *N*-acetylglucosaminidase; PycA, pyruvate carboxylase; PurD, glycine ligase; CysK, cysteine synthase; GlyA, serine hydroxymethyltransferase; GlmS, fructose-6-phosphate aminotransferase; DapA, dihydrodipicolinate synthase. (For interpretation of the references to colour in this figure legend, the reader is referred to the web version of this article.)

The nonvolatile metabolite profiles of both *ldh*-knockout and wild-type LCZ yogurt were characterized using LC-MS-based metabolomics. The total ion chromatograms (TICs) of the QC samples showed strong consistency in both positive and negative modes (Fig. S3), confirming the instrumental stability and analytical reproducibility of the LC-MS system. A total of 3404 non-VOCs were detected in all yogurt samples. PCA revealed six well-defined clusters, indicating distinct metabolic profiles among yogurt samples (Fig. 4A). The clear separation between the SC + LCZ and SC groups indicated significant metabolic alterations induced by LCZ addition, whereas the distinct clustering of SC + Δ*ldh* samples reflected the metabolic effect of *ldh* knockout. Furthermore, the temporal separation between days 1 and 28 in the SC + LCZ, SC + Δ*ldh*, and SC groups revealed substantial metabolic shifts during storage. ANOVA revealed that 1306 compounds differed significantly across the yogurt groups (FDR < 0.05; Fig. 4B). On day 1 (end of fermentation), compared with SC, LCZ addition and *ldh* knockout altered the production of 69 (43 upregulated and 26 downregulated) and 141 (69 upregulated and 72 downregulated) differential metabolites, respectively (|log2FC| > 1, FDR < 0.05; Fig. 4C). After 28 d of storage, substantial metabolic changes were observed in all groups compared to their day 1 profile, with 504 (460 up, 44 down), 458 (396 up, 62 down), and 531 (486 up, 45 down) differential metabolites in the SC, SC + LCZ, and SC + Δ*ldh* groups, respectively. Compared to SC on day 28, supplementation with LCZ and Δ*ldh* induced 191 (26 up, 165 down) and 113 (36 up, 77 down) differential metabolites, respectively. Venn diagram analysis of metabolic changes during storage (day 28 vs. day 1) revealed five distinct clusters (Fig. 4D). Cluster I contained altered metabolites in all three groups, representing common metabolic changes associated with basic starter strains. Cluster II showed metabolites changed in both SC + LCZ and SC + Δ*ldh* groups, reflecting shared responses to starter culture modification. Clusters III and V comprised metabolites uniquely altered in the SC + Δ*ldh* and SC + LCZ groups, indicating the specific effects of *ldh* knockout and LCZ addition, respectively. Cluster VI contained metabolites that were specifically altered in SC, indicating that certain metabolic pathways were uniquely regulated in the original starter culture.

The PLS-DA score plot showed a clear separation among all groups, with Components 1 (18.7%) and 2 (30.6%) explaining 49.3% of the total variance (Fig. 4E). Model quality was confirmed by the high R^2^ (0.994) and Q^2^ (0.824) values. Based on the VIP values (>2) and statistical significance (FDR < 0.05), 62 key metabolites were differentially expressed (Fig. 4F; Table S5). Hierarchical clustering analysis revealed distinct metabolic patterns across different groups, with the SC + Δ*ldh* groups exhibiting the most distinctive profiles on both days 1 and 28. SC + Δ*ldh* yogurts were characterized by significant downregulation of small peptides and amino acids, and marked upregulation of phosphate sugars, organic acids, and carbohydrate-related metabolites.

Pathway analysis using the KEGG pathway database revealed the biological significance of these metabolic alterations and identified the specific metabolic pathways associated with these differential metabolites (Fig. 4G). The most significantly enriched pathway was methane metabolism, followed by glycine, serine, threonine, fructose, and mannose metabolisms. Amino and nucleotide sugar metabolism, glycolysis, and gluconeogenesis showed moderate significance. The analysis identified 17 key metabolites involved in these pathways, including glycerol 3-phosphate, various amino acids (glycine, l-serine, and L-asparagine), and phosphorylated sugars (fructose 6-phosphate and D-mannose 6-phosphate). Organic acids, such as pyruvic, succinic, and acetic acids were identified as important components of these metabolic networks, suggesting substantial changes in energy metabolism and carbon flux distribution.

To further investigate the intricate relationships between these differential metabolites and related genes, particularly *ldh*, a metabolic network was constructed using STITCH with a minimum interaction score threshold of 0.4. The analysis revealed complex interactions between the metabolites (orange nodes) and genes (red nodes), with *ldh* showing the highest degree of connectivity, indicating its central regulatory role. The algorithms employed by STITCH are powerful in identifying chemical-protein interactions based on data from multiple perspectives, including relevant metabolic pathways, molecular crystal structures, results of binding experiments, and available literature; thus, it is advantageous over traditional correlation-based network analysis that does not consider biological information. Network visualization demonstrated that all identified metabolites could interact with *ldh* either directly or indirectly through other genes (such as *DapA*, *GlmS*, and *FbaC*) and intermediary metabolites. Key metabolites, including acetic, malic, and pyruvic acids showed substantial connectivity in the network. These comprehensive interaction patterns strongly suggest that *ldh* knockout triggers a cascade of effects leading to widespread metabolic alterations, thereby significantly influencing yogurt characteristics.

### 3.4 Ldh knockout redirected LCZ metabolic flux from lactic acid to alternative pathways

**Fig. 5:**
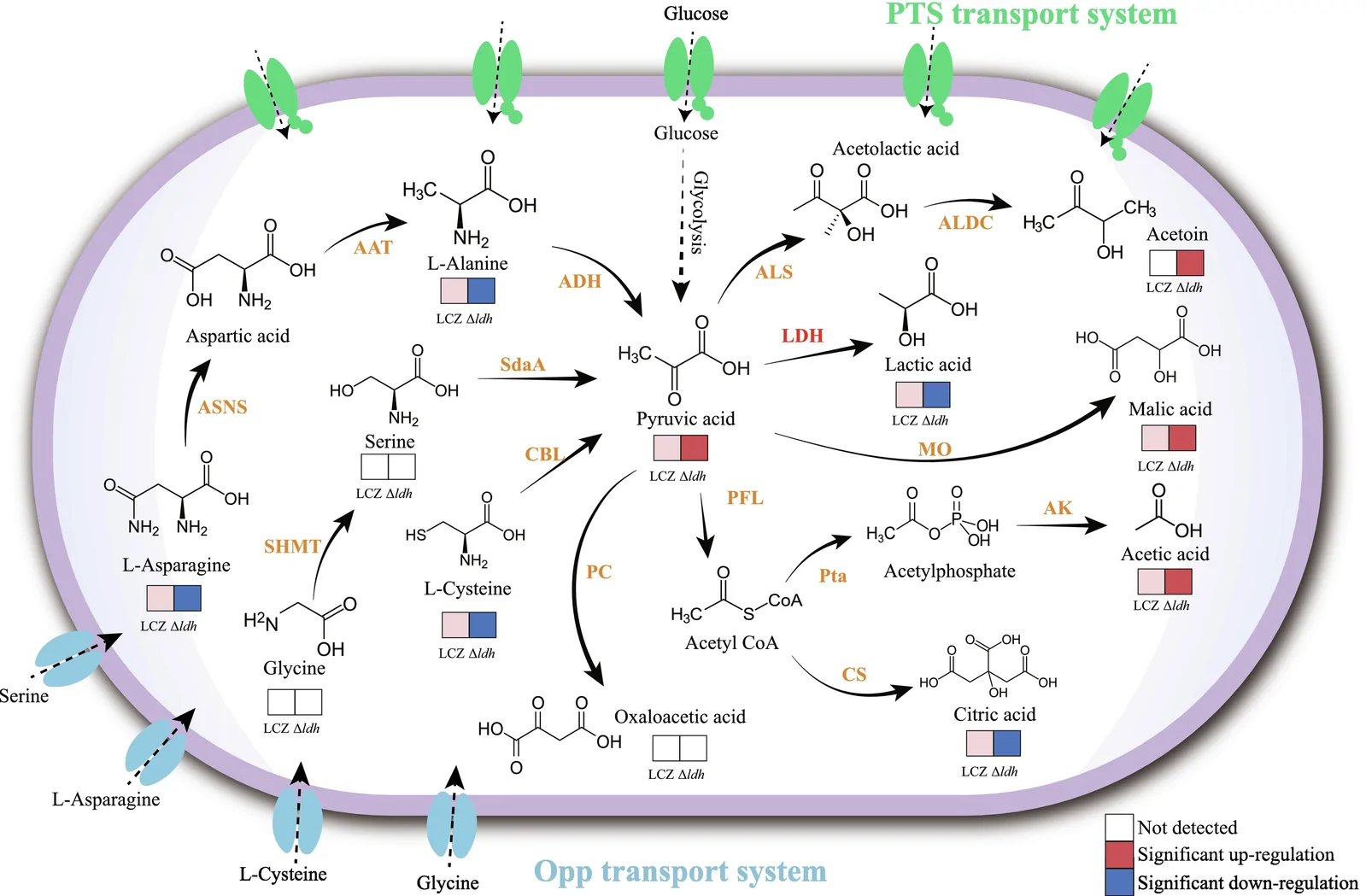
Selected pyruvate-related metabolites mapped to pathway annotations for *Lacticaseibacillus paracasei* Zhang (LCZ). Chemical structures and proposed substrate-uptake systems (PTS in green and Opp in blue) are shown. Colored boxes indicate relative concentration changes in Δ*ldh* versus wild-type LCZ cultures (pink, detected; red, significantly higher; blue, significantly lower; white, not detected). The pathway map was constructed using LCZ annotations in KEGG (https://www.kegg.jp/entry/lcz) and is intended as a pathway-based interpretation rather than a direct measurement of metabolic flux. Abbreviations: ASNS, asparagine synthase; AAT, aspartate aminotransferase; ADH, alanine dehydrogenase; SHMT, serine hydroxymethyltransferase; SdaA, l-serine deaminase; CBL, cystathionine beta-lyase; ALS, acetolactate synthase; ALDC, acetolactate decarboxylase; MO, malate oxidoreductase; PFL, pyruvate-formate lyase; Pta, phosphotransacetylase; AK, acetate kinase; CS, citrate synthase; PC, pyruvate carboxylase. (For interpretation of the references to colour in this figure legend, the reader is referred to the web version of this article.)

Based on the STITCH interaction network and the key compounds identified (acetic, malic, and pyruvic acids) in relation to *ldh*, we constructed a more precise metabolic pathway map using the LCZ genome annotation results from the KEGG database (https://www.kegg.jp/entry/lcz). As shown in Fig. 5, this refined pathway clearly illustrates the upstream and downstream metabolic relationships between *ldh*, lactic acid, and key metabolites in LCZ. Pathway visualization revealed that pyruvic acid is a central metabolic hub that can be generated from glucose via glycolysis and amino acid metabolism via various pathways (e.g., L-asparagine via ASNS-AAT, glycine via SHMT-SdaA, and L-cysteine via CBL). Subsequently, pyruvic acid is converted to lactic acid by *ldh* or channeled into alternative pathways to produce acetoin (via ALS-ALDC), malic acid (via MO), acetic acid (via PFL-Pta-AK), and oxaloacetic acid (via *PC*).

To validate our hypothesis that *ldh* knockout triggers cascade effects that affect both upstream and downstream metabolism, we performed LC-MS/MS-MRM-based targeted metabolomic analysis to determine the absolute changes in metabolite concentrations in the culture medium of wild-type LCZ and Δ*ldh* strains after 24 h of cultivation in MRS medium.

The results showed that compared to wild-type LCZ, the concentrations of lactic acid (45% reduction), citric acid, glycine, serine, L-cysteine, L-asparagine, and L-alanine were significantly decreased in the Δ*ldh* culture medium, whereas the concentrations of pyruvic acid, acetic acid, malic acid, and acetoin were markedly increased (Fig. 5; Fig. S4). Acetoin was exclusively detected in Δ*ldh* strain culture medium at a concentration of 750 ng/mL, while it was undetectable in wild-type LCZ medium.

## 4 Discussion

Increasing consumer awareness about gut health and the role of probiotics in maintaining digestive wellness has significantly driven the global probiotic strains market. According to recent market research, the probiotic yogurt market was valued at USD 2.5 billion in 2022 and is projected to grow at a CAGR of 6.5% between 2023 and 2032 (https://www.gminsights.com/industry-analysis/probiotic-yogurt-market). Despite this promising market outlook, current probiotic yogurt products face a critical challenge of strain homogenization (Gao et al., 2021). Most commercial products primarily rely on conventional starter cultures, such as *Streptococcus thermophilus* and *Lactobacillus delbrueckii* subsp. *bulgaricus*. However, the use of well-documented probiotic strains, such as *Bifidobacterium* and *Lactobacillus* spp., is limited by post-acidification issues during storage, as incorporating multiple probiotic strains can lead to excessive lactic acid production, consequently affecting product shelf life, sensory attributes, and commercial viability (Bai et al., 2020; Li et al., 2024; Peng et al., 2022). Accumulating evidence suggests that yogurt fermented with both starter cultures and probiotics enhances functional properties, including enhanced immunomodulatory effects (Lollo et al., 2013), nutrient bioavailability (Pannerchelvan et al., 2024), and gut health benefits (Abdi-Moghadam et al., 2023; Fernandez & Marette, 2017). These studies highlight the need for innovative strategies and approaches to develop next-generation probiotic yogurt products that can overcome post-acidification challenges while delivering enhanced health benefits.

In this study, we demonstrated the efficacy of Δ*ldh* strain obtained through prophage-derived recombinase-mediated recombineering to alleviate post-acidification in probiotic yogurt products through reduced lactic acid production. Prophage-derived recombinase-mediated recombineering is a highly accurate and efficient method because each step is marker dependent (Wu, Xin, Kong, & Guo, 2021). In contrast to CRISPR-based methods, this approach achieves higher knockout efficiency in *Lactobacillus paracasei* while completely avoiding off-target effects (Song, Huang, Xiong, Ai, & Yang, 2017), which is essential for food-grade probiotic engineering. In this study, a 100% *ldh* knockout efficiency was achieved, which is consistent with previous reports showing 100% mutation efficiency in *Lactobacillus casei* BL23 (Xin, Guo, Mu, & Kong, 2017a). Although this process leaves a mall scar (*loxP72* site, 8 bp) at the modification locus after the selection marker is excised, this scar is extremely short to affect genome function or stability (Campo, Daveran-Mingot, Leenhouts, Ritzenthaler, & Le Bourgeois, 2002).

Knockout of *ldh* significantly altered the metabolic and growth characteristics of LCZ. The *ldh* gene targeted in this study encodes L-lactate dehydrogenase (L-LDH), which catalyzes the conversion of pyruvate to L-lactate while regenerating NAD^+^ from NADH. Deletion of *ldh* would reduce the capacity of LCZ to convert pyruvate into L-lactate, thereby resulting in the slower decline in pH observed for the Δ*ldh* strain during the 48-h cultivation period. Residual acid production was still observed in Δ*ldh* cultures at 37 °C, indicating that acid production was not completely abolished. This residual acidification may be attributable to alternative acid-producing pathways, including the activity of the D-lactate dehydrogenase and malate dehydrogenase encoded in the LCZ genome (https://www.kegg.jp/entry/lcz) and the production of other organic acids. However, the behavior of the Δ*ldh* strain during yogurt storage differed markedly from that observed at 37 °C. At 4 °C, the activities of these alternative metabolic routes were likely strongly suppressed and were insufficient to compensate for the loss of the major L-LDH-mediated lactate-production pathway. Consequently, it did not generate sufficient additional acid to induce significant post-acidification. In contrast, wild-type LCZ retained L-LDH-mediated residual lactate-forming capacity, thereby providing an additional source of acidification in the SC + LCZ group. Thus, the reduced post-acidification of SC + Δ*ldh* yogurt can be attributed primarily to the removal of the dominant L-lactate-producing pathway, together with the limited low-temperature contribution of alternative acid-producing routes.

Our previous study revealed that LCZ could not metabolize lactose and that inoculation of milk with LCZ alone failed to induce coagulation (Zhang et al., 2010). In this study, yogurt fermentation experiments were conducted by combining LCZ with commercial starter cultures (SC). The addition of LCZ to SC significantly enhanced the acidification process, reducing fermentation time by approximately 0.5 h compared to SC alone. However, this synergistic effect leads to post-acidification problems during the storage period. Over the 28-d storage period, yogurt containing LCZ (SC + LCZ) exhibited substantial acid accumulation, with the pH decreasing to 4.15 and TA significantly increased to approximately 98°T. This excessive acidification is disadvantageous for product quality, as the optimal pH range for yogurt sensory attributes is 4.3–4.5, with pH values below 4.1 likely resulting in excessive sourness and off-flavor development (Matela, Pillai, & Thamae, 2019), thereby compromising consumer acceptability. The addition of Δ*ldh* to SC showed comparable fermentation efficiency to SC alone, while maintaining more stable pH and TA values similar to SC during storage, suggesting that *ldh* knockout effectively addressed the post-acidification issue without compromising the fermentation process.

To explore the effect of *ldh* knockout on yogurt flavor, VOCs were analyzed using SPME-GC–MS. The findings revealed distinct VOC profiles between the SC + Δ*ldh* and SC groups, as well as between the SC + LCZ and SC groups during storage. Compared to SC, SC + Δ*ldh* maintained a relatively stable VOC profile, with significant increases observed only in acetoin, decanoic acid, and dodecanoic acid levels. These changes were particularly beneficial, as acetoin and decanoic acid contribute to desirable buttery notes (Cui, Wang, Zheng, & Chen, 2022; Iradukunda, Aida, Ouafi, Barkouch, & Boussaid, 2018), whereas dodecanoic acid imparts creamy characteristics (Tamura et al., 2021), collectively may enhance the typical dairy flavor profile of yogurt. Conversely, after 28 d of storage, SC + LCZ exhibited substantial alterations in its aroma profile, which could potentially compromise the characteristic flavor of commercial yogurt. Specifically, compounds contributing to buttery and fatty notes, which are essential for typical dairy flavor, were significantly downregulated. In contrast, the abundance of aromatic compounds associated with cheese, fatty, floral, and fruity notes was markedly upregulated. However, such drastic shifts in the established commercial yogurt flavor profile can negatively affect consumer acceptability and product marketability. Together, these findings show that the Δ*ldh* strain was associated with a relatively stable volatile profile during storage and with increased relative abundances of acetoin, decanoic acid, and dodecanoic acid. In contrast, the SC + LCZ group exhibited more extensive changes in volatile compounds after 28 d of storage. These results indicate that *ldh* deletion altered the volatile and organic acid profiles of yogurt; however, they do not directly demonstrate improved flavor, sensory quality, or consumer acceptance.

No descriptive sensory evaluation or consumer acceptance test was performed in the present study. Because the yogurt was produced using a genetically edited strain, human sensory evaluation would require appropriate ethical and regulatory approval, which was not obtained for this study. Therefore, the observed metabolomic changes should be interpreted as evidence of altered metabolite profiles and potentially modified flavor characteristics rather than direct sensory improvement. Future studies should evaluate the sensory consequences of the Δ*ldh* strain after the relevant ethical and regulatory requirements have been met.

Non-volatile metabolite profiling revealed that adding LCZ or Δ*ldh* to SC reshaped the yogurt biochemical profile, resulting in distinct metabolic patterns. Specifically, Δ*ldh* induced marked shifts in amino acid and carbohydrate metabolism, as evidenced by the notable downregulation of various peptides and amino acids, along with increased levels of phosphate sugars, organic acids, and other carbohydrate-related metabolites. The reduced peptide content could potentially contribute to a milder taste profile and enhance nutrient absorption (Mirzapour-Kouhdasht, McClements, Taghizadeh, Niazi, & Garcia-Vaquero, 2023). Elevated levels of organic acids, including acetic, succinic, and malic acids, may enhance yogurt flavor complexity and preservation qualities while contributing to the characteristic range and depth of flavor of the product (Y.-H. Yuan et al., 2024). Furthermore, acetic and succinic acids can permeate the intestinal epithelium and be absorbed into circulation, where they perform various functions. They signal by stimulating endocrine and immune cells, influencing metabolic processes, immune responses, and energy homeostasis (Li, Ye, Chen, & Zhang, 2024; Nadjsombati et al., 2018). These metabolic alterations reflect the broader effects of *ldh* modification on cellular metabolism and may contribute to changes in the organic acid profile and potential sourness characteristics of yogurt. *LDH* catalyzes the reduction of pyruvate to lactate while oxidizing NADH to NAD^+^, a process crucial for cellular energy generation and NAD^+^/NADH balance maintenance (Neves et al., 2002). Genetic modifications that disrupt NAD^+^/NADH homeostasis can significantly affect cell growth and survival (Shimizu & Matsuoka, 2019). To minimize these adverse effects, microorganisms employ adaptive strategies through dynamic modulation of cofactor levels and metabolic pathways in response to redox perturbations (Ishii et al., 2007; Long & Antoniewicz, 2014; Long, Gonzalez, Sandoval, & Antoniewicz, 2016).

This metabolic adaptation was validated by a reference standard-based targeted metabolomic analysis, in which Δ*ldh* strains showed altered flux distributions through multiple pathways (Fig. 5): 1) de novo activation of acetoin biosynthesis through the *ALS*-*ALDC* pathway (absent in wild-type), providing a novel route for pyruvate utilization; 2) upregulation of citric, acetic, and malic acid production through the *PLF-CS*, *PLF-Pta-AK*, and *MO* pathways, respectively, suggesting enhanced TCA cycle activity; and 3) enhanced catabolism of amino acids, such as L-alanine, L-cysteine, and L-asparagine, which were funneled into pyruvic acid, indicating strategic nitrogen‑carbon co-utilization to sustain metabolic equilibrium. These coordinated metabolic adjustments reflect the capacity of the Δ*ldh* strain to maintain metabolic homeostasis through activation of alternative pathways when lactic acid production is disrupted.

Our study has certain limitations. The findings demonstrated a distinct multi-pathway adaptation mechanism in LCZ, providing valuable insights for future metabolic engineering of lactic acid bacteria. However, the effects of *ldh* knockout differ among lactic acid bacteria. For instance, *ldh* deletion in *Lacticaseibacillus paracasei* K2003 boosted antimicrobial compound synthesis (Haryani et al., 2024), whereas in *Lactobacillus plantarum*, it affected peptidoglycan precursor synthesis (Ferain et al., 1996) and increased ethanol production (Liu, Nichols, Dien, & Cotta, 2006). Therefore, future studies should investigate the species-specific metabolic rewiring mechanisms following *ldh* deletion to optimize metabolic engineering strategies.

Furthermore, we showed that *ldh* deletion effectively mitigated the post-acidification issues in probiotic yogurt and was associated with altered volatile and non-volatile metabolite profiles. However, several critical aspects require further investigation. First, the long-term genetic stability of Δ*ldh* during industrial-scale fermentation should be evaluated. Second, comprehensive safety assessments are necessary to confirm nontoxicity and regulatory compliance. Third, the probiotic functionality of Δ*ldh*, including gut colonization, immune modulation, and microbiome interactions, remains to be completely characterized. Furthermore, this metabolic engineering approach could be applied beyond yogurt production to other fermented dairy and non-dairy products, thereby broadening its potential commercial applications.

## 5 Conclusion

This study demonstrated that deletion of *ldh* in the probiotic strain LCZ reduced yogurt post-acidification when co-fermented with commercial starters. The engineered strain may also modify the flavor and functional properties of yogurt through altered production of flavor-related compounds (acetoin, decanoic acid, and dodecanoic acid) and metabolites with potential bioactivity (acetic and succinic acids). Mechanistically, *ldh* deletion redirected the carbon flux into the TCA cycle toward the acetic acid, malic acid, and activated acetoin biosynthesis pathways. This deletion also enhanced amino acid catabolism (L-alanine, L-cysteine, and L-asparagine) toward pyruvic acid, achieving nitrogen–carbon co-utilization for metabolic homeostasis. This study provides an innovative strategy for incorporating probiotic strains into fermented dairy products and offers valuable insights for the development of next-generation high-quality health-promoting yogurt products through metabolic engineering.

## CRediT authorship contribution statement

**Bohai Li:** Writing – original draft, Software, Methodology, Data curation, Conceptualization. **Longxiang Ye:** Validation, Methodology, Investigation. **Rong Dai:** Investigation. **Xin Wang:** Formal analysis. **Yongfu Chen:** Writing – review & editing, Supervision.

## Declaration of competing interest

The authors declare that they have no known competing financial interests or personal relationships that could have appeared to influence the work reported in this paper.

## Data Availability

Data will be made available on request.
