# Supplementary material for: Metabolic engineering of Lacticaseibacillus paracasei alleviates post-acidification and reshapes metabolite profiles in probiotic yogurt
Source: Food Chem X. 2026 Sep 3;39:104386. doi: 10.1016/j.fochx.2026.104386 (PMC13571733; doi:10.1016/j.fochx.2026.104386)
Supplement: Supplementary file 1 [file mmc1.docx]

**Supplementary Material**

**Fig. S1.** Overlay total ion chromatograms (TIC) of all samples analyzed by GC-MS.

**Fig. S2.** The number of significantly changed volatile organic compounds (VOCs) at the end of fermentation (Day 1) and after storage (Day 28). A) Results of the Kruskal-Wallis test on VOCs intensity across all groups at Day 1. B) results of the Kruskal-Wallis test on VOCs intensity at Day 28. Each point represents a compound; red dots indicate VOCs with statistically significant changes, while black dots represent VOCs with non-significant changes.

**Fig. S3.** Overlay total ion chromatograms (TIC) of all samples run in the A) positive and B) negative ion modes analyzed by LC-MS.

**Fig. S4.** The absolute changes of pyruvic acid, lactic acid, acetoin, acetic acid, citric acid, malic acid, L-Alanine, L-Asparagine, and L-Cysteine in the culture medium of wild-type LCZ and Δ*ldh* strains after 24 h of cultivation. Statistically significant differences between groups were evaluated using the Wilcoxon test.

**Table S1** Nucleotide sequences of lactate dehydrogenase gene (*ldh*) identified in *Lacticaseibacillus paracasei* Zhang genome

| Gene name | Sequence 5’→3 |
| --- | --- |
| *ldh* | gtggcaagtattacggataaggatcaccaaaaagttattctcgttggtgacggcgccgttggttcaagttatgcctacgcaatggttttgcaaggtatcgctcaggaaatcggaatcgttgacattttcaaggacaagacaaagggtgacgcgattgacttgagcaacgcgctcccattcacaagtcctaagaagatttattcagctgaatacagcgatgctaaggatgctgatctggttgttatcacagctggcgctcctcagaagcctggcgaaactcgtttggacttggttaacaagaacttgaagatcttgaagtccattgttgacccaatcgttgattccggctttaacggtattttcttagttgctgccaacccagttgacattttgacctatgcaacttggaaactttctggcttcccgaagaaccgggttgttggttccggtacttcactggacaccgcccgcttccgtcagtccattgctgaaatggttaatgttgacgctcgttcggtccacgcttacatcatgggcgaacatggtgacactgaattccctgtatggtcccacgctaacattggtggcgttaccatcgctgaatgggttaaggctcatccagaaatcaaggaagacaagcttgttaagatgtttgaagacgttcgtgacgccgcttatgaaatcatcaaactcaagggtgcgaccttctatggtatcgcaactgcccttgcccggatttcaaaggcaatccttaacgacgaaaatgcggttctgccactttccgtttacatggatggtcaatatggcttgaacgacatctacatcggtaccccagctgtgatcaaccgtaatggtatccagaacatcctggaaatcccattgaccgatcacgaagaagaatccatgcagaaatctgcttctcaattgaagaaggttctgaccgatgctttcgctaagaatgacatcgaaactcgtcagtaa |

**Table S2** Oligonucleotides used in this study

| Oligonucleotide | Sequence 5’→3 |
| --- | --- |
| catF | gccactggagcacgtttaaac |
| catR | ccgggctgtaccgttcgtata |
| *ldh*-uF | ctcatcgctgccagtggca |
| *ldh*-uR | taaacgtgctccagtggcgcatgcaaactgcatttcacgttg |
| *ldh*-dF | atacgaacggtacagcccggacgatcactgataagtaagcaaccag |
| *ldh*-dR | gtgcgcttgtgacagttgtcg |

**Table S3**. Multiple reaction monitoring (MRM) transitions and optimized mass spectrometry parameters for key metabolites

| Compound | MRM transition  (m/z) | ESI mode | Declustering  potential  (V) | Collision energy  (eV) |
| --- | --- | --- | --- | --- |
| L-Asparagine | **133.02> 73.45** | POS | 12 | 23 |
|  | 133.02 > 86.56 | POS |  | 15 |
|  | 133.02 > 115.86 | POS |  | 13 |
| L-Alanine | **90.43 >45.06** | POS | 10 | 13 |
| L-cysteine | **122.2>59.2** | POS | 17 | 29.7 |
|  | 122.2>76 | POS |  | 17.04 |
|  | 122.2>86.9 | POS |  | 17.19 |
| Pyruvic acid  (Derivatization) | **357.08 > 136.92** | NEG | -20 | -27 |
|  | 357.08 > 149.78 | NEG |  | -27 |
|  | 357.08 > 177.93 | NEG |  | -24 |
|  | 357.08 > 41.83 | NEG |  | -62 |
|  | 357.08 > 203.92 | NEG |  | -26 |
|  | 357.08 > 121.86 | NEG |  | -26 |
|  | 357.08 > 91.82 | NEG |  | -31 |
| Lactic acid  (Derivatization) | **223.90 > 151.80** | NEG | -20 | -21 |
|  | 223.90 > 136.90 | NEG |  | -26 |
|  | 223.90 > 105.90 | NEG |  | -26 |
| Acetoin  (Derivatization) | **222.10 > 137.02** | NEG | -15 | -20 |
|  | 222.10 > 204.09 | NEG |  | -20 |
| L-Malic acid | **132.87 > 114.87** | NEG | -15 | -20 |
|  | 132.87 > 70.98 | NEG |  | -23 |
|  | 132.87 > 72.70 | NEG |  | -18 |
|  | 132.87 > 89.11 | NEG |  | -16 |
| Acetic acid  (Derivatization) | **193.80 > 151.80** | NEG | -15 | -20 |
|  | 193.80 > 136.90 | NEG |  | -24 |
|  | 193.80 > 175.80 | NEG |  | -20 |
|  | 193.80 > 121.90 | NEG |  | -26 |
|  | 193.80 > 46.00 | NEG |  | -40 |
| Citric acid  (Derivatization) | **595.83 > 221.99** | NEG | -20 | -27 |
|  | 595.83 > 401.15 | NEG |  | -40 |
| Oxaloacetic acid  (Derivatization) | **536.13 > 246.90** | NEG | -25 | -34 |
|  | **536.13 > 246.90** | NEG |  | -27 |
|  | 536.13 > 357.10 | NEG |  | -38 |
|  | 536.13 > 203.97 | NEG |  | -30 |
|  | 536.13 > 383.09 | NEG |  | -34 |
|  | 536.13 > 219.07 | NEG |  | -39 |
|  | 536.13 > 178.04 | NEG |  | -15 |
| Serine | **104.02 > 73.91** | NEG | -10 | -13 |
|  | 104.02 > 72.02 | NEG |  | -12 |
| Glycine | **73.94 > 44.90** | NEG | -25 | -32 |
|  | 73.94 > 32.20 | NEG |  | -30 |

* Bold: quantitative transitions; Regular: qualitative transitions.

**Table S4.** Detailed information of 38 key differential metabolites identified by GC-MS and data analyses

| **Name** | **Molecular**  **weight (Da)** | **Formula** | **RI** | **CAS** | **Class** | **Odor** |
| --- | --- | --- | --- | --- | --- | --- |
| β-Ionone | 192.151 | C13H20O | 1457 | 14901-07-6 | Terpenoids | fruity |
| Propanoic acid | 74.037 | C3H6O2 | 676 | 79-09-4 | Organic acid and Its derivatives | sour |
| Phenol | 94.042 | C6H6O | 980.699 | 108-95-2 | Benzene and substituted derivatives | tarry |
| Octanal | 128.12 | C8H16O | 1003.276 | 124-13-0 | Aldehyde,Ketones,Esters | fruity |
| Nonanal | 142.136 | C9H18O | 1105.027 | 124-19-6 | Aldehyde,Ketones,Esters | fruity |
| Hexanoic acid | 116.084 | C6H12O2 | 987.2008 | 142-62-1 | Organic acid and Its derivatives | fatty |
| Heptanoic acid | 130.099 | C7H14O2 | 1079.914 | 111-14-8 | Organic acid and Its derivatives | cheese |
| Heptanal | 114.104 | C7H14O | 901.3041 | 111-71-7 | Aldehyde,Ketones,Esters | fatty |
| Dodecanoic acid | 214.193 | C13H26O2 | 1481 | 111-82-0 | Aldehyde,Ketones,Esters | creamy |
| Dodecanal | 184.183 | C12H24O | 1402 | 112-54-9 | Aldehyde,Ketones,Esters | floral |
| Decanoic acid | 200.178 | C12H24O2 | 1395.638 | 110-38-3 | Aldehyde,Ketones,Esters | buttery |
| Decanal | 156.151 | C10H20O | 1206.429 | 112-31-2 | Aldehyde,Ketones,Esters | floral |
| Coumarin | 146.037 | C9H6O2 | 1447.63 | 91-64-5 | Heterocyclic compounds | sweet |
| Benzyl alcohol | 108.058 | C7H8O | 1034.764 | 100-51-6 | Alcohol and amines | floral |
| Acetophenone | 120.058 | C8H8O | 1067.823 | 98-86-2 | Aldehyde,Ketones,Esters | fruity |
| Acetoin | 88.052 | C4H802 | 882 | 513-86-0 | Aldehyde,Ketones,Esters | buttery |
| Acetic acid | 60.021 | C2H4O2 | 610 | 64-19-7 | Organic acid and Its derivatives | sour |
| 3-Phenylpropanol | 136.089 | C9H12O | 1233.495 | 122-97-4 | Alcohol and amines | fruity |
| 3-Hexanol | 102.104 | C6H14O | 780 | 623-37-0 | Alcohol and amines | fruity |
| 3-Heptanone | 114.104 | C7H14O | 887 | 106-35-4 | Aldehyde,Ketones,Esters | fatty |
| 2-Undecenal | 168.151 | C11H20O | 1311 | 2463-77-6 | Aldehyde,Ketones,Esters | fruity |
| 2-Tridecanone | 198.198 | C13H26O | 1495.819 | 593-08-8 | Aldehyde,Ketones,Esters | buttery |
| 2-Pentylfuran | 136.089 | C9H12O | 1048 | 20992-69-2 | Heterocyclic compounds | roasted |
| 2-Octenal | 126.104 | C8H14O | 1057.364 | 2548-87-0 | Aldehyde,Ketones,Esters | fatty |
| 2-Octen-1-ol | 128.12 | C8H16O | 1067 | 22104-78-5 | Alcohol and amines | fatty |
| 2-Nonanone | 142.136 | C9H18O | 1091.462 | 821-55-6 | Aldehyde,Ketones,Esters | fruity |
| 2-Nonanol | 144.151 | C9H20O | 1099.2 | 628-99-9 | Alcohol and amines | floral |
| 2-methylheptanoic acid | 144.115 | C8H16O2 | 1109 | 1188-02-9 | Organic acid and Its derivatives | buttery |
| 2-Hexanol | 102.104 | C6H14O | 799.7914 | 626-93-7 | Alcohol and amines | fruity |
| 2-Heptanone | 114.104 | C7H14O | 890.6578 | 110-43-0 | Aldehyde,Ketones,Esters | fruity |
| 2-Decanone | 156.151 | C10H20O | 1151 | 693-54-9 | Aldehyde,Ketones,Esters | fatty |
| 2,4-Undecadienal | 166.136 | C11H18O | 1319 | 13162-46-4 | Hydrocarbons | fatty |
| 2,4-Octadienal | 124.089 | C8H12O | 1021 | 30361-28-5 | Aldehyde,Ketones,Esters | fatty |
| 2,4-Nonadienal | 138.104 | C9H14O | 1120 | 6750-03-4 | Aldehyde,Ketones,Esters | buttery |
| 2(5H)-Furanone | 84.021 | C4H4O2 | 912.8259 | 497-23-4 | Aldehyde,Ketones,Esters | buttery |
| 1-Hexanol | 130.136 | C8H18O | 1028.648 | 104-76-7 | Alcohol and amines | floral |
| 1-Decanol | 158.167 | C10H22O | 1271.529 | 112-30-1 | Alcohol and amines | fatty |
| 13-Octadecenal | 266.261 | C18H34O | 2007 | 58594-45-9 | Aldehyde,Ketones,Esters | waxy |

**Table S5.** Detailed information of 62 key differential metabolites identified by LC-MS and data analyses

| **Name** | **Formula** | **Molecular weight (Da)** | **RT**  **(min)** | **CAS** | **HMDB** | **Class** |
| --- | --- | --- | --- | --- | --- | --- |
| 3-Dehydroquinic acid | C7H10O6 | 190.0477 | 0.93 | 10534-44-8 | HMDB0012710 | Organic acid and Its derivatives |
| 3-Oxoglutaric acid | C5H6O5 | 146.0215 | 1.27 | 542-05-2 | HMDB0013701 | Organic acid and Its derivatives |
| 9-Decenoic acid | C10H18O2 | 170.1307 | 6.70 | 14436-32-9 | HMDB0031003 | Oxidized lipids |
| Acetic acid | C2H4O2 | 60.02110 | 0.78 | 64-19-7 | HMDB0000042 | Organic acid and Its derivatives |
| Aconitic acid | C6H6O6 | 174.0164 | 1.79 | - | HMDB0247961 | Organic acid and Its derivatives |
| Acoric acid | C15H24O4 | 268.1675 | 6.23 | 55700-39-5 | HMDB0038165 | Organic acid and Its derivatives |
| ADP | C10H15N5O10P2 | 427.0294 | 1.29 | 58-64-0 | HMDB0001341 | Nucleotide and Its metabolites |
| ADP-glucose | C16H25N5O15P2 | 589.0822 | 0.95 | 2140-58-1 | HMDB0006557 | Nucleotide and Its metabolites |
| Ala-Asn-Asp-Asp | C15H23N5O10 | 433.1445 | 0.86 | - | - | Small Peptide |
| Ala-Ile-Ala | C12H23N3O4 | 273.1689 | 1.55 | - | - | Small Peptide |
| Ala-Ile-Tyr | C18H27N3O5 | 365.1951 | 2.24 | - | - | Small Peptide |
| Arg-Asn-Asn | C14H26N8O6 | 402.1975 | 1.69 | - | - | Small Peptide |
| Arg-Tyr-Phe | C24H32N6O5 | 484.2434 | 2.33 | - | - | Small Peptide |
| Ascorbic acid | C6H8O6 | 176.0321 | 1.13 | 50-81-7 | HMDB0000044 | CoEnzyme and vitamins |
| Asp-Ile-Ala-Lys | C19H35N5O7 | 445.2536 | 2.96 | - | - | Small Peptide |
| Asp-Tyr | C13H16N2O6 | 296.1008 | 1.21 | 22840-03-5 | HMDB0028765 | Small Peptide |
| beta-N-Acetylglucosamine | C8H15NO6 | 221.0899 | 0.69 | 14131-68-1 | HMDB0000803 | Sugar derivatives |
| Biotin | C10H16N2O3S | 244.0882 | 3.27 | 58-85-5 | HMDB0000030 | CoEnzyme and vitamins |
| Citric acid | C6H8O7 | 192.0270 | 0.90 | 77-92-9 | HMDB0000094 | Organic acid and Its derivatives |
| D-Glycerate 3-phosphate | C3H7O6P | 169.9980 | 1.02 | 591-59-3 | HMDB0001112 | Sugar derivatives |
| D-Mannose 1-phosphate | C6H13O9P | 260.0297 | 0.59 | 27251-84-9 | HMDB0006330 | Phosphate sugars |
| D-Mannose 6-phosphate | C6H13O9P | 260.0297 | 0.85 | - | HMDB0304312 | Phosphate sugars |
| Fructose 6-phosphate | C6H13O9P | 260.0297 | 2.47 | 643-13-0 | HMDB0000124 | Phosphate sugars |
| Fucose 1-phosphate | C6H13O8P | 244.0348 | 1.21 | 16562-59-7 | HMDB0001265 | Carbohydrates and Its metabolites |
| Gln-Tyr-Glu | C19H26N4O8 | 438.1751 | 1.48 | - | - |  |
| Glucosamine 6-phosphate | C6H14NO8P | 259.0457 | 0.60 | - | HMDB0001254 | Phosphate sugars |
| Glyceraldehyde 3-phosphate | C3H7O7P | 185.9929 | 1.05 | 3443-58-1 | HMDB0060180 | PA |
| Glyceric acid | C3H6O4 | 106.0266 | 1.02 | 473-81-4 | HMDB0000139 | Organic acid and Its derivatives |
| Glycerol 3-phosphate | C3H9O6P | 172.0137 | 1.04 | 17989-41-2 | HMDB0000126 | GP |
| Glycerophosphoric acid | C3H9O6P | 172.0137 | 0.88 | - | HMDB0252849 | PA |
| Glycine | C2H5NO2 | 75.0320 | 1.82 | 56-40-6 | HMDB0000123 | Amino acids |
| Gly-Pro-Leu | C13H23N3O4 | 285.1689 | 1.93 | 47066-32-0 | - | Small Peptide |
| Gly-Tyr-Tyr-Ser-Lys | C29H40N6O9 | 616.2857 | 3.32 | - | - | Small Peptide |
| Homovanillic acid | C9H10O4 | 182.0579 | 2.59 | 306-08-1 | HMDB0000118 | Organic acid and Its derivatives |
| Ile-Ala-Trp | C20H28N4O4 | 388.2111 | 7.06 | - | - | Small Peptide |
| Ile-Asp-Lys | C16H30N4O6 | 374.2165 | 2.09 | - | - | Small Peptide |
| Ile-Leu-Asn-Asp | C20H35N5O8 | 473.2486 | 3.92 | - | - | Small Peptide |
| Indoleacetic acid | C10H9NO2 | 175.0633 | 2.38 | 87-51-4 | HMDB0000197 | Organic acid and Its derivatives |
| Lactose 6-phosphate | C12H23O14P | 422.0825 | 0.72 | - | HMDB0006789 | Organic acid and Its derivatives |
| L-Asparagine | C4H8N2O3 | 132.0535 | 1.30 | 70-47-3 | HMDB0000168 | Amino acids |
| L-Phenylalanine | C9H11NO2 | 165.0790 | 2.02 | 63-91-2 | HMDB0000159 | Amino acids |
| L-Serine | C3H7NO3 | 105.0426 | 1.41 | 56-45-1 | HMDB0000187 | Amino acids |
| Lys-Asp-Leu-Glu | C21H37N5O9 | 503.2591 | 3.41 | - | - | Small Peptide |
| Maleylacetoacetic acid | C8H8O6 | 200.0321 | 2.45 | 5698-52-2 | HMDB0002052 | Organic acid and Its derivatives |
| Malic acid | C4H6O5 | 134.0215 | 8.90 | 97-67-6 | HMDB0000156 | Amino acid derivatives |
| Mannitol 1-phosphate | C6H15O9P | 262.0454 | 1.19 | 15806-48-1 | HMDB0001530 | Phosphate sugars |
| Met-Ile-Thr | C15H29N3O5S1 | 363.1828 | 2.82 | - | - | Small Peptide |
| myo-Inositol 6-phosphate | C6H13O9P | 260.0297 | 1.03 | 15421-51-9 | HMDB0002985 | Organic acid and Its derivatives |
| N-Acetyl-D-glucosamine | C8H15NO6 | 221.0899 | 0.83 | 7512-17-6 | HMDB0000215 | Sugar derivatives |
| N-Acetylmannosamine | C8H15NO6 | 221.0899 | 1.25 | 1136-44-3 | HMDB0001129 | Sugars |
| Phenylethylamine | C13H16N2O4 | 264.1110 | 1.48 | 28047-15-6 | HMDB0006344 | Amino acid derivatives |
| Phosphoenolpyruvate | C3H5O6P | 167.9824 | 0.97 | 138-08-9 | HMDB0000263 | Phosphoric acids |
| Pro-Trp-Trp | C27H29N5O4 | 487.2220 | 1.81 | - | - | Small Peptide |
| Pyruvic Acid | C3H4O3 | 88.0160 | 0.83 | 127-17-3 | HMDB0000243 | Organic acid and Its derivatives |
| Shikimic Acid | C7H10O5 | 174.0528 | 1.17 | 138-59-0 | HMDB0003070 | Organic acid and Its derivatives |
| Sorbitol-6-phosphate | C6H15O9P | 262.0454 | 2.38 | 20479-58-7 | HMDB0005831 | Phosphate sugars |
| Succinic acid | C4H6O4 | 118.0266 | 1.50 | 110-15-6 | HMDB0000254 | Organic acid and Its derivatives |
| Trehalose 6-phosphate | C12H23O14P | 422.0825 | 0.72 | 4484-88-2 | HMDB0001124 | Phosphate sugars |
| Trp-Gln-Arg | C22H32N8O5 | 488.2496 | 2.37 | - | - | Small Peptide |
| Uracil | C4H4N2O2 | 112.0273 | 1.07 | 66-22-8 | HMDB0000300 | Nucleotide and Its metabolites |
| Val-Ser-Ala-Lys | C17H33N5O6 | 403.2431 | 2.30 | - | - | Small Peptide |
| Xylulose 5-phosphate | C5H11O8P | 230.0192 | 1.17 | 4212-65-1 | HMDB0000868 | Phosphate sugars |
